# A systematic approach to study the pH-dependent release, productivity and product specificity of dextransucrases

**DOI:** 10.1186/s12934-019-1208-8

**Published:** 2019-09-10

**Authors:** Jonas Schmid, Julia Bechtner, Rudi F. Vogel, Frank Jakob

**Affiliations:** Lehrstuhl für Technische Mikrobiologie, Technische Universität München (TUM), Freising, Germany

**Keywords:** Lactic acid bacteria, Dextransucrase, Systematic approach, Extracellular enzyme

## Abstract

**Background:**

Dextransucrases are extracellular enzymes, which catalyze the formation of α-1→6-linked glucose polymers from sucrose. These enzymes are exclusively expressed by lactic acid bacteria, which commonly acidify the extracellular environment due to their physiology. Dextransucrases are thus confronted with steadily changing reaction conditions in regards to the environmental pH, which can further affect the amount of released dextransucrases. In this work, we studied the effect of the environmental pH on the release, the productivity and the product specificity of the dextransucrase expressed by *Lactobacillus* (*L.*) *hordei* TMW 1.1822. Dextransucrases were recovered as crude extracts at pH 3.5–pH 6.5 and then again used to produce dextrans at these pH values. The respectively produced dextran amounts and sizes were determined and the obtained results finally systematically correlated.

**Results:**

Maximum dextran amounts were produced at pH 4.0 and pH 4.5, while the productivity of the dextransucrases significantly decreased at pH 3.5 and pH 6.5. The distribution of dextran amounts produced at different pH most likely reflects the pH dependent activity of the dextransucrases released by *L. hordei*, since different transglycosylation rates were determined at different pH using the same dextransucrase amounts. Moreover, similar hydrolysis activities were detected at all tested conditions despite significant losses of transglycosylation activities indicating initial hydrolysis prior to transglycosylation reactions. The molar masses and rms radii of dextrans increased up to pH 5.5 independently of the stability of the enzyme. The gelling properties of dextrans produced at pH 4.0 and pH 5.5 were different.

**Conclusions:**

The presented methodological approach allows the controlled production of dextrans with varying properties and could be transferred and adapted to other microbes for systematic studies on the release and functionality of native sucrases or other extracellular enzymes.

## Introduction

Dextrans contain α–1→6-linked glucose monomers in their backbone, which can be branched at positions *O2*, *O3* or *O4.* They are synthesized by extracellular dextransucrases (EC 2.4.1.5) belonging to the family 70 of glycoside hydrolases (GH 70) [1–5]. These enzymes use the energy of the glycosidic bond of their substrate sucrose for the polymerization of glucose, while fructose is continuously released [2, 6–8]. Moreover, they can hydrolyze sucrose if water is used as acceptor instead of the growing polymer chain. Dextrans are exclusively expressed by lactic acid bacteria (LAB) living in various ecological niches. The properties as well as the biosynthesis of dextran have been extensively studied in the past due to its wide use in biotechnological applications [4, 5, 9–14]. The biological functions of dextran are not completely revealed, but there are many studies concerning the role of dextran in biofilm formation [15–19]. As LAB are naturally subjected to pH fluctuations, changes in pH during growth are crucial factors influencing the release of dextransucrases as well as their activity [20–23] implying the complexity of natural dextran formation. The aim of this study was to establish a systematic approach, which gives new insights into the pH-dependent release, productivity and product specificity of dextransucrases and, beyond that, enables an improved control over the formation of specific dextran fractions. For this purpose, we used the native dextransucrase of *Lactobacillus hordei* TMW 1.1822, which is efficiently released in the presence of sucrose and serves as sucrose converting enzyme in the extracellular environment [24–26].

## Materials and methods

### Strain, media and cultivation

The strain *L. hordei* TMW 1.1822 isolated from water kefir was cultivated in modified MRS as described before [27], containing 10 g/L of glucose and 10 g/L fructose, 10 g/L peptone from casein, 5 g/L yeast extract, 5 g/L meat extract, 4 g/L K_2_HPO_4_, 2.6 g/L KH_2_HPO_4_, 3 g/L NH_4_Cl, 1 g/L Tween 80, 0.5 g/L cysteine-HCl, 0.2 g/L MgSO_4_∙7H_2_O, 0.036 g/L MnSO_4_ H_2_O and 15 g/L agar for solid media. The pH of MRS was adjusted to 6.2 and cultivations were generally performed at 30 °C. To obtain cells in the mid-exponential growth phase main cultures were inoculated with 4% (v/v) of 30 h grown pre-cultures diluted to an OD_590nm_ = 2.0. All experiments were carried out in biological triplicates. To exclude any possible contamination during storage at − 80 °C, prior to the experimental start the strain identity was verified with matrix-assisted laser-desorption-ionization time-of-flight mass spectrometry (MALDI-TOF-MS) by comparing the generated mass spectra with our in house database.

### Dextran production and isolation

**Fig. 1:**
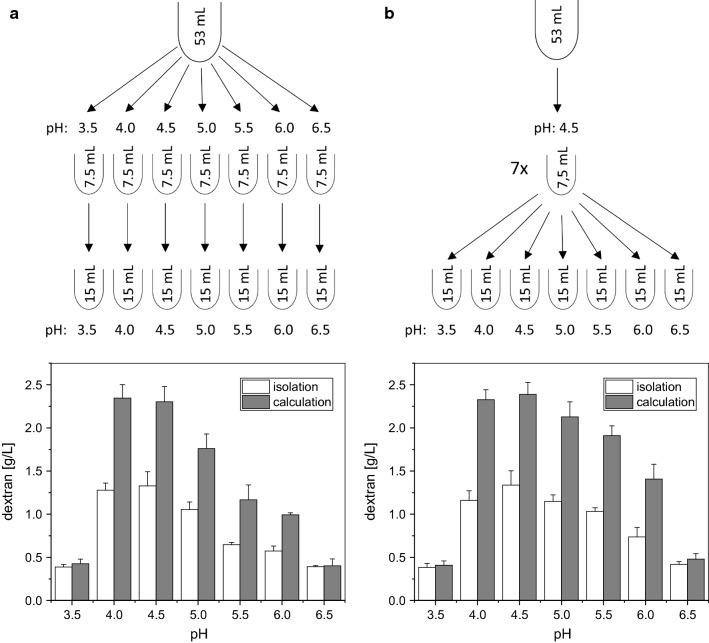
Overview of the experimental setups used to characterize dextran production at different pH. Cells were grown in 53 mL mMRS medium (without sucrose) for 18 h, split up to 7.5 mL samples, centrifuged and re-dissolved in 7.5 mL buffer (0.1 M Na_2_HPO_4_/0.1 M citric acid + 0.1 M sucrose) of the desired pH values, respectively. In setup (**a**), dextransucrase containing supernatants were collected at pH 3.5 to 6.5, while in setup (**b**) the release pH for all samples was kept constant at pH 4.5. After 3.0 h of incubation in these buffers, the cells were removed by centrifugation and the supernatants were sterile-filtered. 7.5 mL buffer of the required pH to reach the final production pH were added, followed by 24 h of incubation and subsequent dextran quantification. Bars indicate the produced dextran amounts in g/L, which were either determined gravimetrically (isolated dextran) or via calculation of the totally produced amount using the calculated transglycosylation activity (24 h). Data are expressed with mean ± SD of three biological replicates

Figure 1 depicts the procedure for recovery of dextransucrase containing solutions. At first, the grown main culture (53 mL) was split in 7.5 mL tubes. Cells were then removed by centrifugation at 10,000×*g* for 10 and re-dissolved in equal volumes (7.5 mL) of 0.1 M citrate–phosphate buffers [12] containing 0.1 M sucrose. After 3.0 h of incubation at 30 °C the cells were removed by centrifugation (10,000×*g*, 10 min) and sterile filtration (0.2 µm nylon filters, Phenomenex, Germany). The pH of the acidified buffer was re-adjusted to the desired value by adding 7.5 mL of buffer of calculated pH values to reach the final dextran production pH according to McIlvaine [28]. The release of the enzyme for 3.0 h in presence of 0.1 M sucrose was either performed at pH values from 3.5 to 6.5 (steps of 0.5 units) or at pH 4.5 for all samples. Subsequently, the EPS were synthesized at 30 °C for 24 h at pH values of 3.5, 4.0, 4.5, 5.0, 5.5, 6.0, 6.5. Enzymatic reactions were stopped by adding two volumes (15 mL) of chilled ethanol for dextran isolation or by shock freezing (− 20 °C) in case of subsequent HPLC analysis (2.4), respectively. The ethanol treated buffer solutions were stored over night at 4 °C. Precipitated dextrans were then recovered by centrifugation at 10,000×*g* for 10 min and subsequently redissolved in an appropriate amount of water. The water polysaccharide mixture was dialyzed against dH2O using dialysis tubing (SERVAPOR, SERVA Electrophoresis GmbH, Germany) with a MWCO (molecular weight cut off) of 3.5 kDa at 4 °C and subsequently lyophilized for at least 24 h. The isolated dextran was quantified by weighing.

### Verification of the released dextransucrase

The protein composition of the samples was analyzed by vertical SDS-PAGE, carried out in a Mini-PROTEAN^®^ Tetra Cell Electrophoresis System (Bio-Rad laboratories, Hercules, USA). A separation gel (12% (w/v)) total monomer concentration with a stacking gel (4% (w/v)) were used. Prior to application on the gel, protein samples were diluted in 2× Laemmli buffer (Sigma-Aldrich, St. Louis, USA) and denatured at 90 °C for 10 min. Separation was initially started at 80 V, 10 min and continued at 100 V for 120 min using a Power Pack 3000 unit (Bio-Rad laboratories, Hercules, USA). Visualization of proteins was conducted by silver staining as described by Blum, Beier [29]. For determination of the size of the dextransucrases and identification of the corresponding SDS-PAGE lane, an activity staining was performed. Therefore, following electrophoresis, SDS gels were washed three times for 10 min in sodium acetate buffer (20 mM, 0.3 mM CaCl_2_, 0.1% Tween 80, pH 5.4) at 4 °C and incubated in the same buffer, containing 5% (w/v) sucrose, at 30 °C overnight. Afterwards, the gels were washed in an aqueous solution containing 50% (v/v) methanol and 10% (v/v) acetic acid for 30 min, followed by washing in deionized water (dH_2_O) for 30 min. For oxidization of formed dextrans, the gels were incubated in periodic acid solution [1% (w/v) periodic acid (≥ 99.0%, Sigma-Aldrich, St. Louis, USA), 3% (v/v) acetic acid] for 45 min and subsequently washed in dH_2_O for 1 h. Finally, dextrans were stained by Schiff’s reagent (Sigma-Aldrich, St. Louis, USA) until discrete pink bands appeared. The gels were washed in dH_2_O for 5 min. If not otherwise stated, all steps were carried out at room temperature. Total protein concentrations of the buffer supernatants were determined by the Coomassie/Bradford Protein Assay Kit (Thermo Fisher Scientific, USA). The assay was performed in standard 96 microtiter plates according to the instructions of the manufacturer.

### Quantification of sugars

For quantification of free sugars, high pressure liquid chromatography (HPLC) (Dionex Ultimate 3000, Thermo Fisher Scientific, USA) coupled to refractive index detection (ERC Refractomax 521, Thermo Fisher Scientific, USA) was used. Sugar separations were performed on a Rezex RPM-Monosaccharide Pb^2+^ column (Phenomenex Ltd., Germany) using water as mobile phase and a flow rate of 0.6 mL/min at 85 °C. The sugars were identified and quantified using sugar standards and subsequently generated calibration curves and the Chromeleon™ software (Version 6.8, Dionex, Germany).

### Analysis of dextran sizes

The characterization of molecular weights and rms radii of the isolated dextrans was accomplished by asymmetric flow field-flow fractionation (AF4) coupled with multi-angle laser light scattering (MALS) (Dawn Heleos II, Wyatt Technology, Germany) analysis and UV detection (Dionex Ultimate 3000, Thermo Fisher Scientific, USA). The freeze dried dextran was at first redissolved in 0.05 M NaNO_3_ (which also served as the eluent) at a final concentration of 0.1 mg/mL. 100 µL of the respective sample were then injected into the separation channel, equipped with a 10 kDa cellulose membrane. Separations were performed using a detector-flow rate of 1 mL/min and a cross-flow gradient of 3 to 0.1 mL/min over 15 min, followed by 15 min of a steady cross flow of 0.1 mL/min. All chromatograms were analyzed with the software ASTRA 6.1 (Wyatt Technologies, Germany) using a dn/dc value of 0.1423 mL/g [30] and the Berry model integrated in the ASTRA software. The distributions of rms radii were calculated in the particle mode, whereas the molar mass distributions were determined by using the respective UV concentration signals of the dextrans at 400 nm [20, 23, 25]. The extinction coefficients of dextrans were determined in standard 96 microtiter plates at a wavelength of 400 nm (SPECTROstar, BMG LABTECH) according to the Beer–Lambert law.

### Statistical analysis

Data were evaluated with SigmaPlot Version 12.5 (Systat Software GmbH, Germany) by one-way ANOVA. Differences in the means were considered as significant for p < 0.05 and are depicted in Additional file 1.

## Results

### Quantification of dextrans produced at different pH

**Fig. 2:**
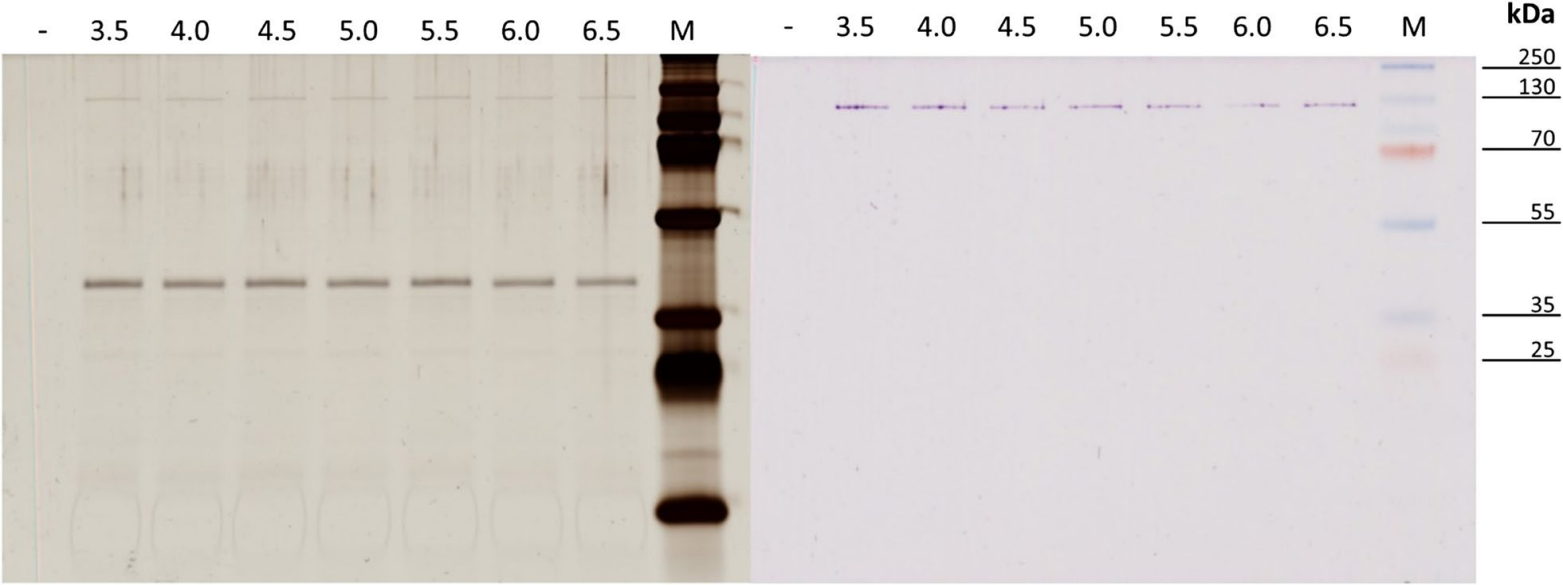
Silver (left) and activity (right) stained SDS-gels of the buffer supernatants obtained after 3 h of incubation of *Lactobacillus hordei* TMW 1.1822 at different pH (setup A); negative control (−), marker (M)

For recovery of native dextransucrases and subsequent production of dextran at different pH, two different experimental setups were conducted (Fig. 1). As *L. hordei* TMW 1.1822 releases its dextransucrase only in the presence of sucrose [24], pre-cultured cells (mMRS medium) were at first re-suspended and incubated in sucrose-supplemented buffers. After 3 h of incubation in these buffers, the respective cell densities, pH values as well as the released and consumed sugars were determined. While the viable cells remained constant, a reduction in pH and the release of fructose and glucose were observed (Table 1). Afterwards, cells were separated and the dextransucrase containing supernatants were used to produce dextran at different pH for 24 h. The respective pHs were adjusted to the final production pH according to McIlvaine [28]. The produced dextrans were quantified both by weighing of isolated dextrans as well as by calculation of the total dextran amount (per L) using the equation c (transglycosylated glucose) × 162.16 g/mol (molar mass of glucose in dextran) (2.4). To visualize the crude protein extracts and to verify the presence of the dextransucrase, SDS-PAGE and subsequent silver and activity staining were performed using the supernatants recovered in setup A (Fig. 2). The dextransucrase band was similarly intense at all conditions and similar total protein concentrations were present in the tested supernatants (Table 1). In both setups, maximum dextran amounts were produced at pH 4.0 and 4.5. The lowest amounts were determined for pH 3.5 and pH 6.5. Outside of the depicted pH range, no dextran production took place, respectively (data not shown). Dextran amounts were significantly higher at pH 5.5 and 6.0 in setup B (Fig. 1b; Additional file 1), while at other pH similar amounts were produced in both approaches. The loss of dextran during the isolation process accounts for ~ 40% in between pH 4.0–pH 6.0 in both setups. At pH 3.5 and 6.5 no differences in the calculated and the isolated amounts were detectable (Fig. 1a, b). The concentration of liberated glucose, which reflects the hydrolysis activity of the dextransucrase, was similar at all test conditions (~ 12 mM glucose/27 h) (Table 1; Additional file 1).

**Table 1:** Log10 CFU/mL, pH and glucose, fructose concentrations after 3 h of dextransucrase release

	Smp./pH	Log10 CFU/mL 3 h	pH 3 h	Glucose 3 h [mM]	Fructose 3 h [mM]	Glucose 27 h [mM]	Fructose 27 h [mM]	Protein [µg/mL]
Setup A	3.5	9.69 ± 0.17	3.37 ± 0.01	4.84 ± 0.02	4.90 ± 0.07	12.00 ± 0.29	14.69 ± 0.04	16.38 ± 2.11
	4	9.61 ± 0.03	3.88 ± 0.01	4.91 ± 0.08	8.41 ± 0.19	12.07 ± 0.43	30.04 ± 1.00	16.39 ± 1.87
	4.5	9.63 ± 0.13	4.38 ± 0.00	5.03 ± 0.05	8.50 ± 0.32	12.18 ± 1.02	29.86 ± 0.07	16.40 ± 1.90
	5	9.64 ± 0.09	4.77 ± 0.01	5.04 ± 0.03	7.77 ± 0.36	12.16 ± 1.29	25.74 ± 0.52	16.27 ± 1.98
	5.5	9.71 ± 0.10	5.25 ± 0.01	4.72 ± 0.13	7.07 ± 0.30	11.34 ± 0.72	20.88 ± 1.73	15.83 ± 2.02
	6	9.62 ± 0.10	5.73 ± 0.01	5.22 ± 0.13	7.27 ± 0.11	11.77 ± 0.62	19.81 ± 0.75	17.12 ± 2.00
	6.5	9.67 ± 0.05	6.21 ± 0.00	4.89 ± 0.03	5.83 ± 0.20	11.78 ± 0.02	15.21 ± 0.51	16.60 ± 1.88
Setup B	3.5		4.37 ± 0.01	4.84 ± 0.07	8.40 ± 0.14	11.86 ± 0.32	17.94 ± 0.35	
	4		4.37 ± 0.01	4.93 ± 0.02	8.58 ± 0.22	12.04 ± 0.37	30.04 ± 0.51	
	4.5		4.37 ± 0.01	5.03 ± 0.10	8.44 ± 0.54	12.35 ± 0.26	30.49 ± 0.85	
	5		4.37 ± 0.01	5.16 ± 0.06	8.28 ± 0.46	12.40 ± 0.97	28.66 ± 0.73	
	5.5		4.37 ± 0.01	4.99 ± 0.13	8.56 ± 0.57	11.64 ± 0.82	26.99 ± 0.37	
	6		4.37 ± 0.01	4.93 ± 0.09	7.90 ± 0.41	12.06 ± 0.69	23.71 ± 1.09	
	6.5		4.37 ± 0.01	4.71 ± 0.08	8.27 ± 0.87	11.79 ± 0.20	18.24 ± 0.22	

For calculation of the totally produced dextran amounts, glucose and fructose concentrations were additionally determined at the end of dextran production (27 h). Sugar concentrations were determined according to 2.4. The protein concentrations of the buffer supernatants were determined by the Bradford assay. Data are expressed with mean ± SD of three biological replicates, respectively

*Smp.* sample

### Determination of the molecular size and weight of dextran produced at different pH

**Fig. 3:**
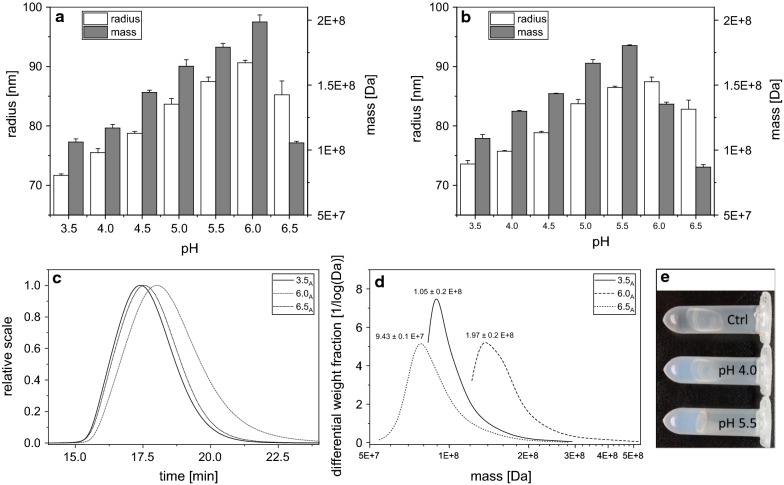
Averaged molar masses [Da] and rms radii [nm] of dextrans produced in experimental setups (**a**, **b**) (Fig. 1). **c** AF4 chromatograms (light scattering signal at 90°) of representative dextrans isolated from setup A. **d** Differential weight fractions of the dextrans depicted in Fig. 2c. Data are expressed as average of three biological replicates with ± SD, respectively. **e** Photographs of dextrans produced at pH 5.5 and pH 4.0 redissolved in a concentration of 5% (w/v) in water; control (ctrl): pure water

The isolated dextrans from both setups were further analyzed using AF4-MALS-UV regarding their molecular weight and rms radii. In both setups, similar tendencies were observed. The rms radii increased from pH 3.5 to 6.0 and decreased at pH 6.5 (Fig. 3a, b). The molecular weight increased to the same extent except for pH 6.0 (Fig. 3a, b). At pH 6.5, the averaged rms radii and molecular weights decreased, respectively. Furthermore, differences in the retention times were observed, as the dextrans exhibiting smaller averaged molar masses and radii eluted earlier (Fig. 3c, d). According to the principles of AF4 separation, it can thus be concluded that the hydrodynamic volumes of these dextrans increase with their molecular size and weight [31, 32] (Fig. 3c, d). Figure 3e shows the gel forming capability of dextrans of predominantly small fractions (pH 4.0) compared to the ones with bigger fractions (pH 5.5). Both dextrans were re-dissolved in a concentration of 50 g/L resulting in increased gel stability for the dextran derived from pH 5.5.

## Discussion

Diverse studies reported about the sucrose-inducible or pH-dependent release of dextransucrases in LAB [22, 33–36]. We recently observed that the dextransucrase of *L. hordei* is released into buffer supernatants efficiently in the presence of sucrose [24]. Within the present study we wanted to *inter alia* verify, if the environmental pH additionally influences the dextransucrase release by *L. hordei*.

At first we observed that maximum dextran amounts were obtained at pH 4.0 and 4.5 in setup A (Fig. 1a), which are within the range of pH values for optimal activities of dextransucrases expressed by some other LAB [37, 38]. However, the respectively used enzyme amounts could have significantly differed in setup A, as the dextransucrases were collected at different pH. Setup B was thus used to see if the tendencies in regard to produced dextran amounts could indeed be driven by differing enzyme concentrations, at which similar overall trends were observed (Fig. 1). At pH 3.5 and pH 6.5 the decrease in produced dextran amounts was similar in both setups (Fig. 1a, b) and the amount of released glucose was similar at all tested pH conditions (Table 1: ~ 12 mM/27 h). Moreover, the total protein concentrations exhibited no significant differences (~ 16 µg/mL; Table 1/Additional file 1) and the dextransucrase recovered at different pH appeared similarly intense (Fig. 2). These findings strongly suggest that similar dextransucrase amounts were present in all assays, also considering that distinctly different numbers of identical dextransucrases could not lose transglycosylation, or rather, keep up hydrolysis activities at similar levels at non optimum pH, respectively. However, comparatively higher amounts of dextrans were produced at pH 5.0, 5.5, 6.0, if the dextransucrases had been recovered at pH 4.5 (setup B). This indicates a less reducing effect concerning the productivity and stability of the dextransucrase due to the inevitable denaturation and the concomitant loss of transglycosylation activity at non optimum pH, if it had been preliminarily recovered working at its approximate optimum pH. Furthermore, the comparable hydrolysis rates observed at all tested pH conditions imply a directed hydrolysis mechanism, which takes place independently of the loss of transglycosylation activity and, thus, most likely before the polymerization reaction.

Our data further reveal that considerable amounts of dextrans could not be isolated at more optimum pH using a standardized EPS isolation approach [39–41], which could be explained by the more frequent occurrence of short-chain dextrans, which get lost during the ethanol precipitation or dialysis of the samples. Therefore, the observed increases in averaged molar masses and rms radii (Fig. 3) with rising pH refer to the respective high molecular weight fractions of the dextrans produced by *L. hordei*. A similar relationship between sizes of sucrase-synthesized EPS and the fermentation pH was reported for levans synthesized by acetic acid bacteria (AAB) [23]. The reasons for the pH dependent mass increases of glucans and fructans still remain unclear. First observations, however, reveal that high molecular weight dextrans produced by *L. hordei* at different pH exhibit different functional properties (Fig. 3e), which coincides with the findings about levans from AAB [23]. A proper control of the pH during the production of diverse sucrase-synthesized EPS hence appears to be a decisive factor for recovery of functionally versatile glucans and fructans. Overall, similar pH dependent size distributions were obtained in both setups (Fig. 3) enabling the reproducible recovery of defined glucans independently of the initial dextransucrase activity, which however can affect the produced dextran amounts as described above. The observed decrease in averaged molar masses at pH 6.0 in setup B despite the increase in rms radii (Fig. 3) is actually not clear and subject of further investigations.

## Conclusions

Our results give new insights into the pH-dependent release, functionality and product specificity of the dextransucrase expressed by *L. hordei*. The presented systematic approach allows the controlled production of dextran with varying properties and could be transferred and adapted to other glucan- or fructansucrase expressing microbes.

## Supplementary information

**Additional file 1.** p-values of all relevant and discussed samples. Empty fields refer to p > 0.05.

## Data Availability

The datasets used and/or analyzed during the current study are available from the corresponding author on reasonable request.

## Abbreviations

AAB: acetic acid bacteria
AF4-MALS-UV: asymmetric flow field-flow fractionation coupled with multi-angle laser light scattering and UV detection
EPS: exopolysaccharide
GH 70: glycoside hydrolase 70 family
HPLC: high-performance liquid chromatography
LAB: lactic acid bacteria
*L.*: *Lactobacillus*
MWCO: molecular weight cut-off
SDS-PAGE: sodium dodecyl sulfate polyacrylamide gel electrophoresis
Smp: sample
TMW: Technische Mikrobiologie Weihenstephan
