# Supplementary figures and images for: A systematic approach to study the pH-dependent release, productivity and product specificity of dextransucrases
Source: Microb Cell Fact. 2019 Sep 10;18:153. doi: 10.1186/s12934-019-1208-8 (PMC6737638; doi:10.1186/s12934-019-1208-8)

## Slide 1
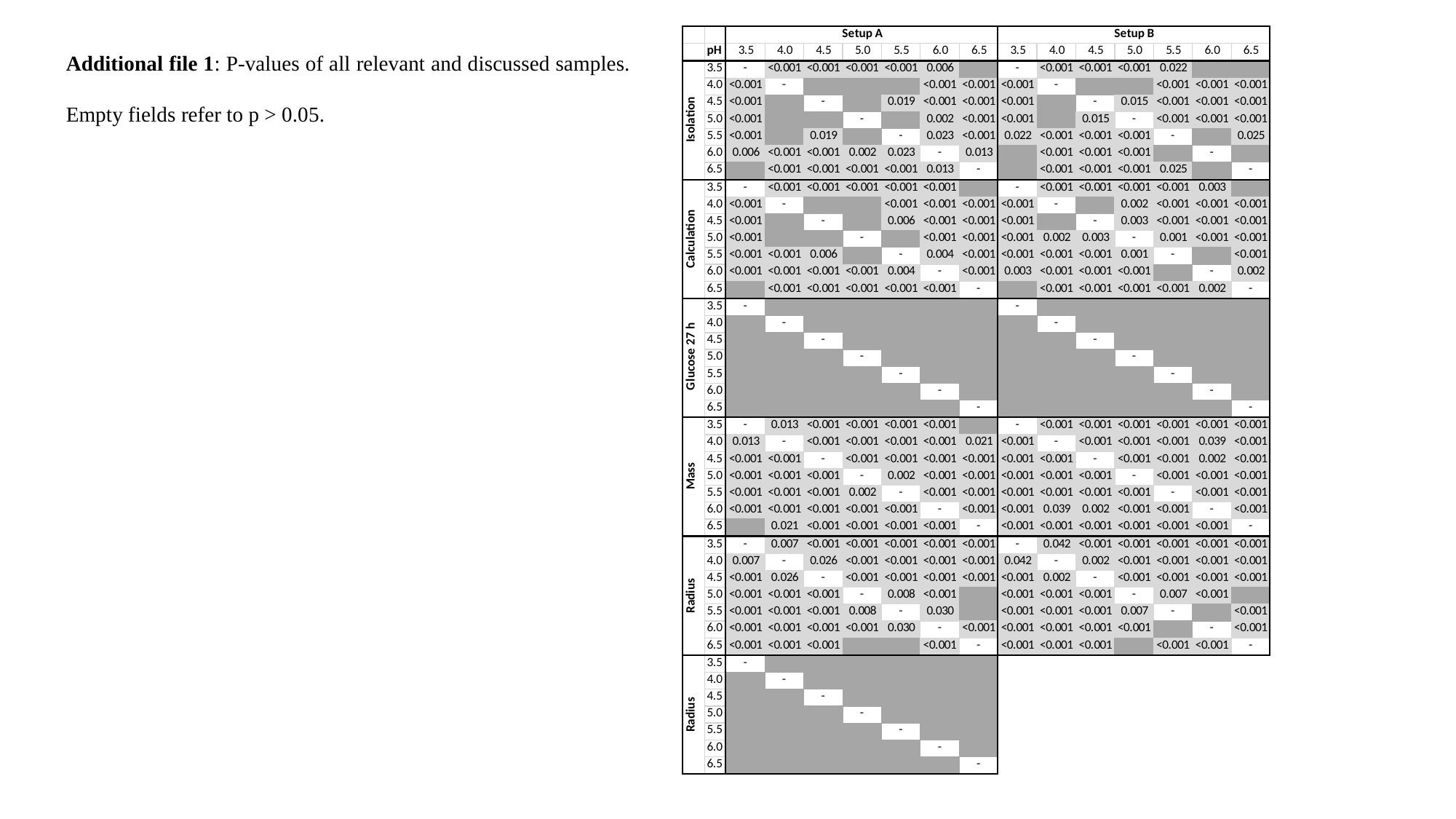

Additional file 1: P-values of all relevant and discussed samples. Empty fields refer to p > 0.05.

Supplement: Supplementary file 1 — Additional file 1. p-values of all relevant and discussed samples. Empty fields refer to p > 0.05. [file 12934_2019_1208_MOESM1_ESM.pptx]
